# The spectrum of tuberculosis described as differential DNA methylation patterns in alveolar macrophages and alveolar T cells

**DOI:** 10.1186/s13148-022-01390-9

**Published:** 2022-12-17

**Authors:** Isabelle Pehrson, Shumaila Sayyab, Jyotirmoy Das, Nina Idh, Jakob Paues, Melissa Méndez-Aranda, César Ugarte-Gil, Maria Lerm

**Affiliations:** 1 Division of Inflammation and Infection, Department of Biomedical and Clinical Sciences, Faculty of Medicine and Health Sciences, Linköping University, Lab 1, Floor 12, 58185 Linköping, Sweden; 2 Bioinformatics Unit (Core Facility), Linköping University, Linköping, Sweden; 3 Clinical Genomics Linköping, SciLife Laboratory, Department of Biomedical and Clinical Sciences, Linköping University, Linköping, Sweden; 4 Division of Infectious Diseases, Department of Biomedical and Clinical Sciences, Faculty of Medicine and Health Sciences, Linköping University, Linköping, Sweden; 5 Laboratorio de Investigación en Enfermedades Infecciosas, Facultad de Ciencias Y Filosofía, Universidad Peruana Cayetano Heredia, Lima, Peru; 6 School of Medicine, Universidad Peruana Cayetano Heredia, Lima, Peru; 7 Instituto de Medicina Tropical Alexander Von Humboldt, Universidad Peruana Cayetano Heredia, Lima, Peru

**Keywords:** DNA methylation, Tuberculosis, Biosignature, Epigenetics, Sputum induction, IGRA

## Abstract

**Background:**

Host innate immune cells have been identified as key players in the early eradication of *Mycobacterium tuberculosis* and in the maintenance of an anti-mycobacterial immune memory, which we and others have shown are induced through epigenetic reprogramming. Studies on human tuberculosis immunity are dominated by those using peripheral blood as surrogate markers for immunity. We aimed to investigate DNA methylation patterns in immune cells of the lung compartment by obtaining induced sputum from *M. tuberculosis-* exposed subjects including symptom-free subjects testing positively and negatively for latent tuberculosis as well as patients diagnosed with active tuberculosis. Alveolar macrophages and alveolar T cells were isolated from the collected sputum and DNA methylome analyses performed (Illumina Infinium Human Methylation 450 k).

**Results:**

Multidimensional scaling analysis revealed that DNA methylomes of cells from the tuberculosis-exposed subjects and controls appeared as separate clusters. The numerous genes that were differentially methylated between the groups were functionally connected and overlapped with previous findings of trained immunity and tuberculosis. In addition, analysis of the interferon-gamma release assay (IGRA) status of the subjects demonstrated that the IGRA status was reflected in the DNA methylome by a unique signature.

**Conclusions:**

This pilot study suggests that *M. tuberculosis* induces epigenetic reprogramming in immune cells of the lung compartment, reflected as a specific DNA methylation pattern. The DNA methylation signature emerging from the comparison of IGRA-negative and IGRA-positive subjects revealed a spectrum of signature strength with the TB patients grouping together at one end of the spectrum, both in alveolar macrophages and T cells. DNA methylation-based biosignatures could be considered for further development towards a clinically useful tool for determining tuberculosis infection status and the level of tuberculosis exposure.

**Supplementary Information:**

The online version contains supplementary material available at 10.1186/s13148-022-01390-9.

## Background

Tuberculosis (TB) is a pulmonary infection and until the COVID-19 pandemic it was the leading cause of death from a single infectious agent [1]. An expansion of the current toolkit for diagnosis, prevention and treatment is critical for reaching the United Nations’ Sustainable Development Goals for 2030 of ending the TB epidemic [2]. TB is caused by *Mycobacterium tuberculosis*, which transmit via aerosols and target alveolar macrophages in exposed individuals [3]. In recent years, the concept of trained immunity has evolved as an epigenetically encoded immune memory that can be triggered by a variety of stimuli and is reflected in a reprogrammed immune state characterized by a higher magnitude of response to subsequent pathogen challenges. The discovery of epigenetically regulated antimicrobial defense mechanisms goes beyond the classical understanding of immune defense and opens a new field of research. Along this line, we have demonstrated that administration of the BCG vaccine to healthy subjects induced profound epigenetic alterations in immune cells, which correlated with enhanced anti-mycobacterial activity in macrophages isolated from the vaccinees [4]. The changes were reflected in the DNA methylome, with the strongest response being recorded within weeks after vaccination [4]. Our observation that BCG induces alterations of the DNA methylome of immune cells has later been confirmed by others [5, 6]. Since BCG vaccination reflects an in vivo interaction between immune cells and viable mycobacteria, we here hypothesized that natural exposure to *M. tuberculosis* would induce similar changes not only in TB patients, but also in individuals who have been exposed to TB. We performed this study to evaluate if alveolar macrophages and alveolar T cells exposed to *M. tuberculosis* undergo epigenetic reprogramming, reflected as differentiated DNA methylation (DNAm) patterns in individuals exposed to the pathogen. Alveolar macrophages and alveolar T cells were procured via induced sputum, which has been verified as an equivalent method to bronchoalveolar lavage for the seclusion of the two cell types [7], and isolated in accordance with a well-established method [8, 9]. Analyses of the DNA methylomes of the isolated alveolar macrophages and alveolar T cells allowed us to identify distinct DNAm signatures in patients with TB and TB-exposed individuals.

Biosignatures of TB have been proposed to lay ground for next generation of diagnostics for TB and accumulating literature have been published studying possible TB biosignatures [10, 11]. DNAm-based biosignatures exists for many other diseases e.g. inflammatory arthritis and asthma [12, 13]. Further, ethnicity and age also influence the DNA methylome [14, 15]. We included patients with tuberculosis, domestic- and occupational TB-exposed subjects and healthy controls. The first part of our pilot study was performed in Linköping, Sweden, which is a low-endemic site for TB. When we found a distinct DNAm signature of TB disease and TB-exposure we expanded the study to include TB patients and TB-exposed in Lima, Peru, a TB high-burden country. Despite the difference in TB prevalence of the two populations of Sweden and Peru it was possible to discern TB exposure based on the DNA methylomes of the subjects. Attempts have been made to identify biomarkers of TB and TB progression, including DNAm events in peripheral immune cells of active and latent TB patients [16, 17], however no study to our knowledge has investigated DNAm in immune cells of the lung compartment of patients with TB and TB-exposed subjects, making this pilot study unique.

## Results

### Subject characteristics

**Fig. 1:**
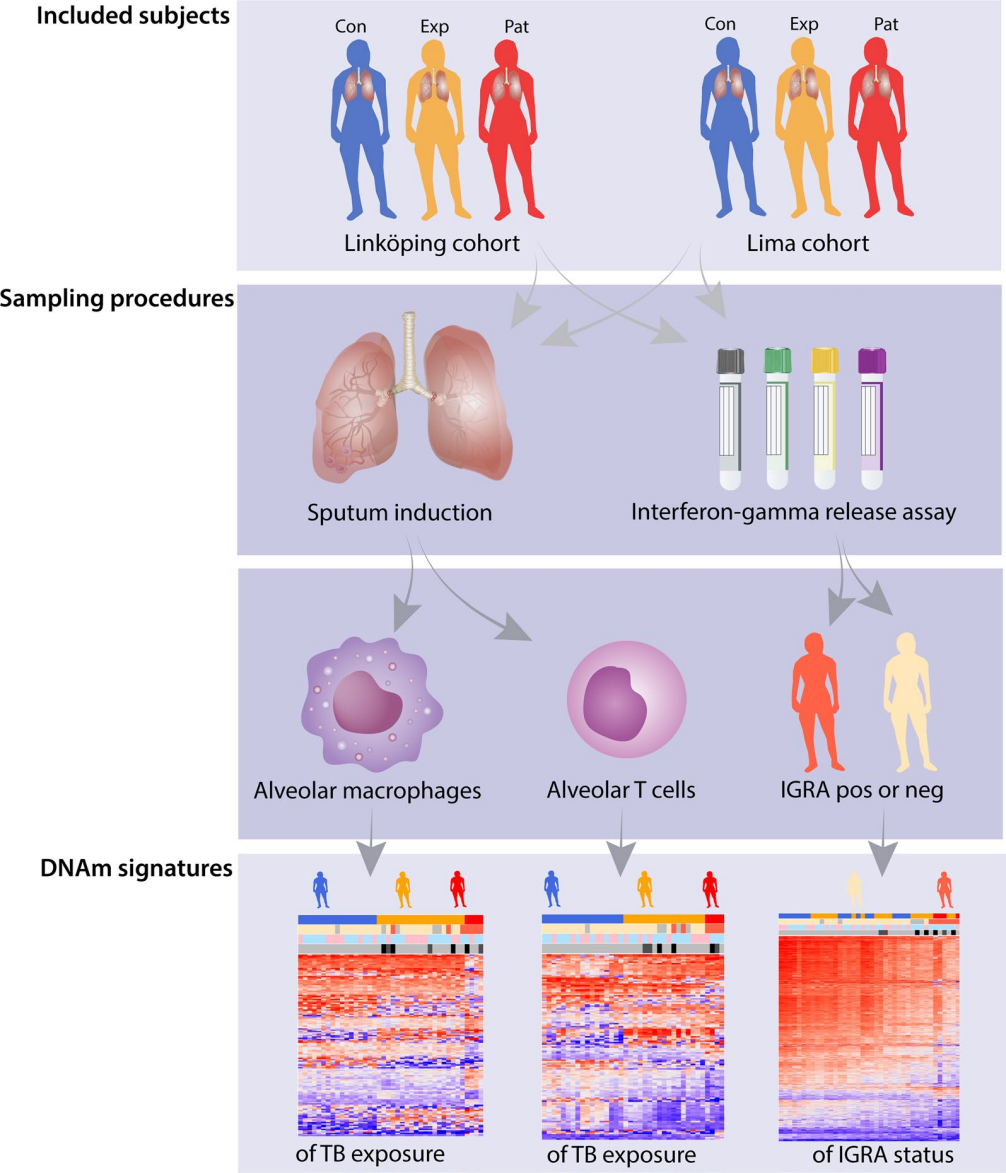
Flow chart of the study design. DNAm, DNA methylation; TB, tuberculosis; Con, control group; Exp, TB-exposed; Pat, TB patients; IGRA, interferon-gamma release assay; pos, positive; neg, negative

Figure 1 represents a flow chart of the study design. Patients with TB (Pat, *n* = 4), TB-exposed (Exp, *n* = 19) and healthy controls (Con, *n* = 18) in Sweden and in Peru (Table 1) were included. The exposed subjects from Peru all had occupational exposure to TB. The TB-exposed from Sweden were close contacts to a TB patient, enrolled via routine contact tracing at the hospital. The healthy control group of Peru and Sweden were healthy students and university employees (Universidad Peruana Cayetano Heredia and Linköping University, respectively) that weren’t aware of previous exposure to TB. The two TB patients included in Lima had drug-resistant TB (MDR and XDR, respectively, (both on > 3 years of treatment) and the two patients included in Linköping had drug-sensitive TB (< 2 months of treatment). Two of the TB patients and all exposed subjects except one had been BCG-vaccinated (Table 1). Interferon-Gamma Release Assay (IGRA) status was determined and among the exposed individuals, three were positive and four had a borderline-positive result (T-SPOT.TB [18]). Among the controls, one subject was classified as ‘borderline’- positive (QuantiFERON-TB® [19]) (Table 1). Subjects with asthma were found among the exposed and controls, but not in the patient group (Table 1). The participants donated induced sputum, from which we isolated HLA-DR-positive alveolar macrophages and CD3 positive alveolar T cells [7, 20]. DNA was prepared from the cells and DNAm data generated. Applying singular value decomposition (SVD), we found no significant differences in the DNAm data between the Pat, Exp and Con groups regarding BMI, smoking, age and sex (Additional file 2: Fig. S1). For asthma, we found significant DNAm differences (Additional file 2: Fig. S1), but we could not identify any differentially methylated CpG sites (DMCs) when comparing the subjects with asthma to those without asthma with the same significance threshold used for TB exposed versus non-exposed (Benjamini-Hochberg (BH)-corrected *p* < 0.01, data not shown). Batch effects, which co-varied with the two cohorts (= country of sampling), were corrected using a singular variance analysis algorithm (SVA) [21]. Among Exp or controls, country of sampling did not reveal any contribution to the variance.

**Table 1:** Demographic characteristic data of the subjects.

Characteristics	Pat (*n* = 4)	Exp (*n* = 19)	Con (*n* = 18)
Country of residence			
Sweden	2 (50%)	5 (26%)	6 (33%)
Peru	2 (50%)	14 (74%)	12 (66%)
Mean age (year)a	27.5 ± 4.7	29.6 ± 9.5	23.5 ± 5.5
Mean height (cm)a	171 ± 6.2	169.2 ± 9.8	170.6 ± 7.7
Mean weight (kg)a	59.3 ± 3.9	70.1 ± 14.9	68.4 ± 14.4
Mean Body Mass Index (BMI)a	20.3 ± 0.7	24.3 ± 3.5	23.4 ± 3.8
Sex			
Male	3 (75%)	11 (58%)	10 (66%)
Female	1 (25%)	8 (42%)	8 (44%)
Smoking			
Current	1 (25%)	3 (16%)	0 (0%)
Previous	1 (25%)	2 (10%)	0 (0%)
Never	2 (50%)	14 (74%)	18 (100%)
BCG			
Vaccinated	2 (50%)	18 (95%)	15 (83%)
Non-vaccinated	2 (50%)	1 (5%)	3 (17%)
IGRA result			
Positive	2 (50%)	3 (16%)	0 (0%)
Negative	0 (0%)	12 (63%)	17 (94%)
ND	2 (50%)	0 (0%)	0 (0%)
Borderline	0 (0%)	4 (21%)	1 (6%)
Asthma			
Asthma	0 (0%)	4 (21%)	4 (22%)
No asthma	4 (100%)	15 (79%)	14 (78%)

Pat, TB patients; Exp, TB-exposed; Con, control group; BMI, body mass index; BCG, bacillus Calmette Guerìn; IGRA, interferon gamma release assay; ND, no data

^a^The standard deviation of the mean values is added to the age, height, weight and BMI

### DNA methylome data from TB-exposed individuals form a separate cluster

**Fig. 2:**
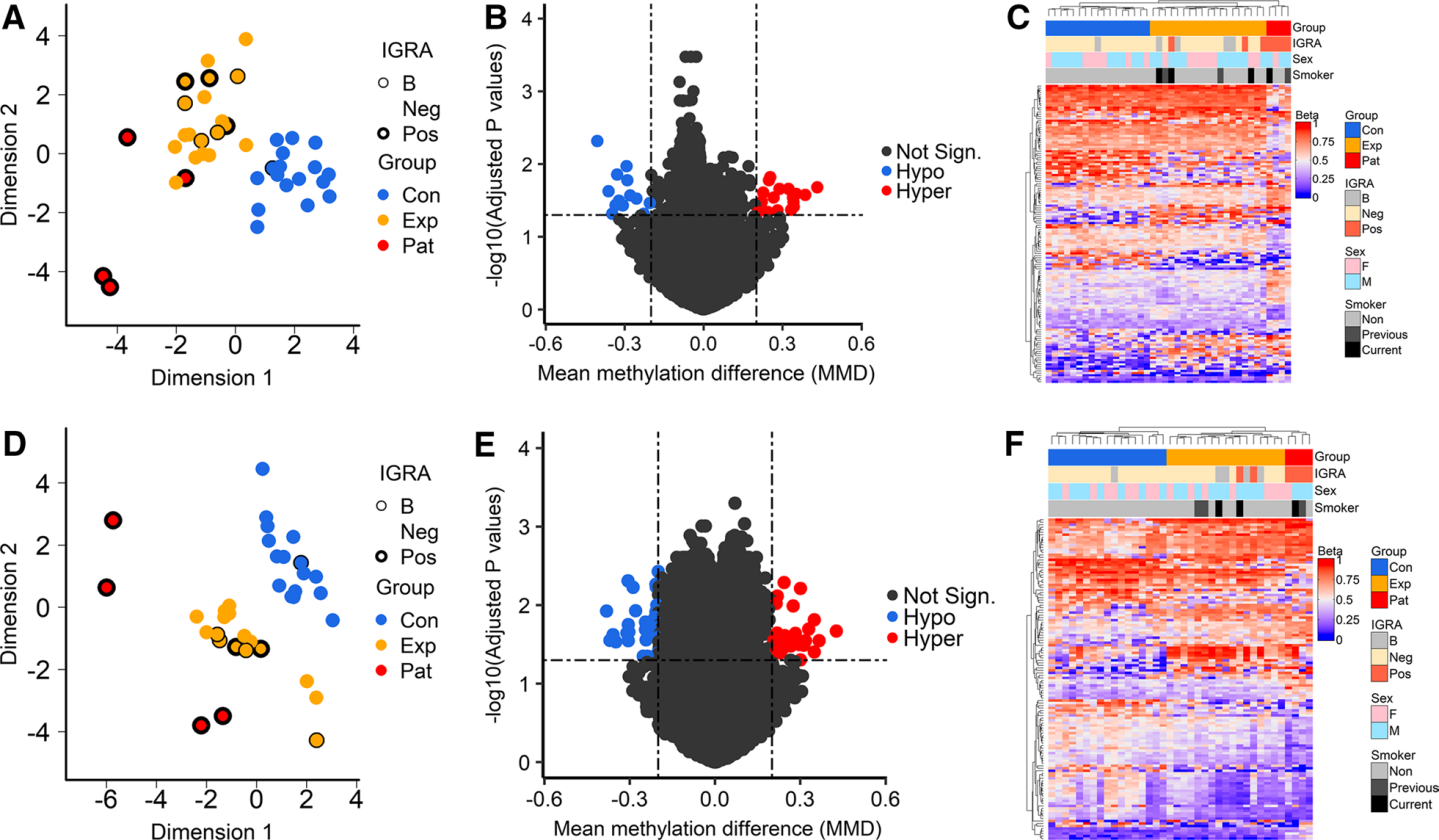
DNAm signatures of TB exposure in alveolar macrophages and alveolar T cells. **A**, **D** Multidimensional scaling (MDS) analyses of controls and TB-exposed/patient samples. MDS plot of dimension 1 (x-axis) and dimension 2 (y-axis) showing the distances between the samples using the top 1000 most variable CpG positions of the alveolar macrophages (**A**) and alveolar T cells (**D**). **B**, **E** EnhancedVolcano plot of the differentially methylated CpG-sites (DMCs) separating the controls from the TB-exposed/patient group in the alveolar macrophages (**B**) and alveolar T cells (**E**). Each dot represents a comparison of mean methylation at an individual CpG site. The x-axis is the mean methylation difference (MMD) with vertical line representing a cut-off MMD >  + -0.2. The y-axis is the negative log_10_ of adjusted *p*-value with the cut off FDR *p*-value of 0.05 shown with dash-dotted horizontal line. Blue CpGs, hypomethylated; red CpGs, hypermethylated. **C**, **F** Heatmap analysis of the alveolar macrophages (**C**) and alveolar T cells (**F**) revealing a distinct DNA methylation pattern of the controls and TB-exposed and patient group. The heatmap is plotted from the absolute β-values of the top DMCs differentiating the groups. Cluster dendrogram is calculated using the Euclidean distance method. DNAm, DNA methylation; TB, tuberculosis; MDS, multidimensional scaling; DMC, differentially methylated CpG-site; MMD, mean methylation difference; FDR, False Discovery Rate; Con, control group; Exp, TB-exposed; Pat, TB patients; F, female; M, male; ND, no data; IGRA, interferon-gamma release assay

The 1 000 most variable positions of the DNAm data were subjected to multidimensional scaling (MDS) to investigate any inherent differences between three study groups (Fig. 2A, alveolar macrophages and 2D, alveolar T cells) using the Euclidean distances between the samples. This analysis revealed distinct differences between the study groups (Con and Exp) in the alveolar macrophages (Fig. 2A) and alveolar T cells (Fig. 2D) on the first dimension, with the TB cases (Pat) clearly distinct from the Con and clustering closely to the Exp.

Next, we identified DMCs and differentially methylated genes (DMGs), *n* = 137 for the alveolar macrophages (Additional file 9: Table S1) and *n* = 128 for the alveolar T cells (Additional file 9: Table S2) by comparing the TB-exposed (Pat and Exp) and control groups (Con) for each cell population. To filter out the most significantly altered DMGs in the dataset, the stringency criteria of mean methylation difference (MMD) > 0.2 and *p *value _BH_ < 0.05 were applied. First, we analyzed the DMCs of the Pat vs Con groups, the Pat vs Exp groups, the Exp vs Con groups and lastly the DMCs between the Pat and Exp groups vs the Con group. For the alveolar macrophages, we found 137 DMCs and for the alveolar T cells we found 128 DMCs, when merging the results of the individual analysis (Pat-Con, Pat-Exp, Exp-Con, (Pat-Exp)-Con) (Fig. 2B, E). The results are depicted as volcano plots, which show that DNA methylomes of TB-exposed and TB patients strongly differ in the alveolar macrophages and alveolar T cells as compared to control subjects (Fig. 2B, E). The DMCs are presented in a heatmap, in which we see a clear separation between the Pat, Exp and Con groups (Fig. 2C, F). One IGRA positive subject clustered with the Con-group, identified as IGRA borderline-positive (Fig. 2A, C, D, F). For the alveolar macrophages and alveolar T cells, the patients with drug-resistant TB formed a separate group that was distinguished from the patients with drug-sensitive TB (Additional files 3 and 4: Fig. S2A and S2B). We found 16 DMCs between the patients with drug-resistant TB and drug-sensitive TB for the alveolar T cells (Additional file 9: Table S3, MMD > 0.2, *p*-value_BH _< 0.1), but no DMCs for the alveolar macrophages (data not shown).

### Pathway enrichment analysis reveals common and unique interactomes in the datasets

**Fig. 3:**
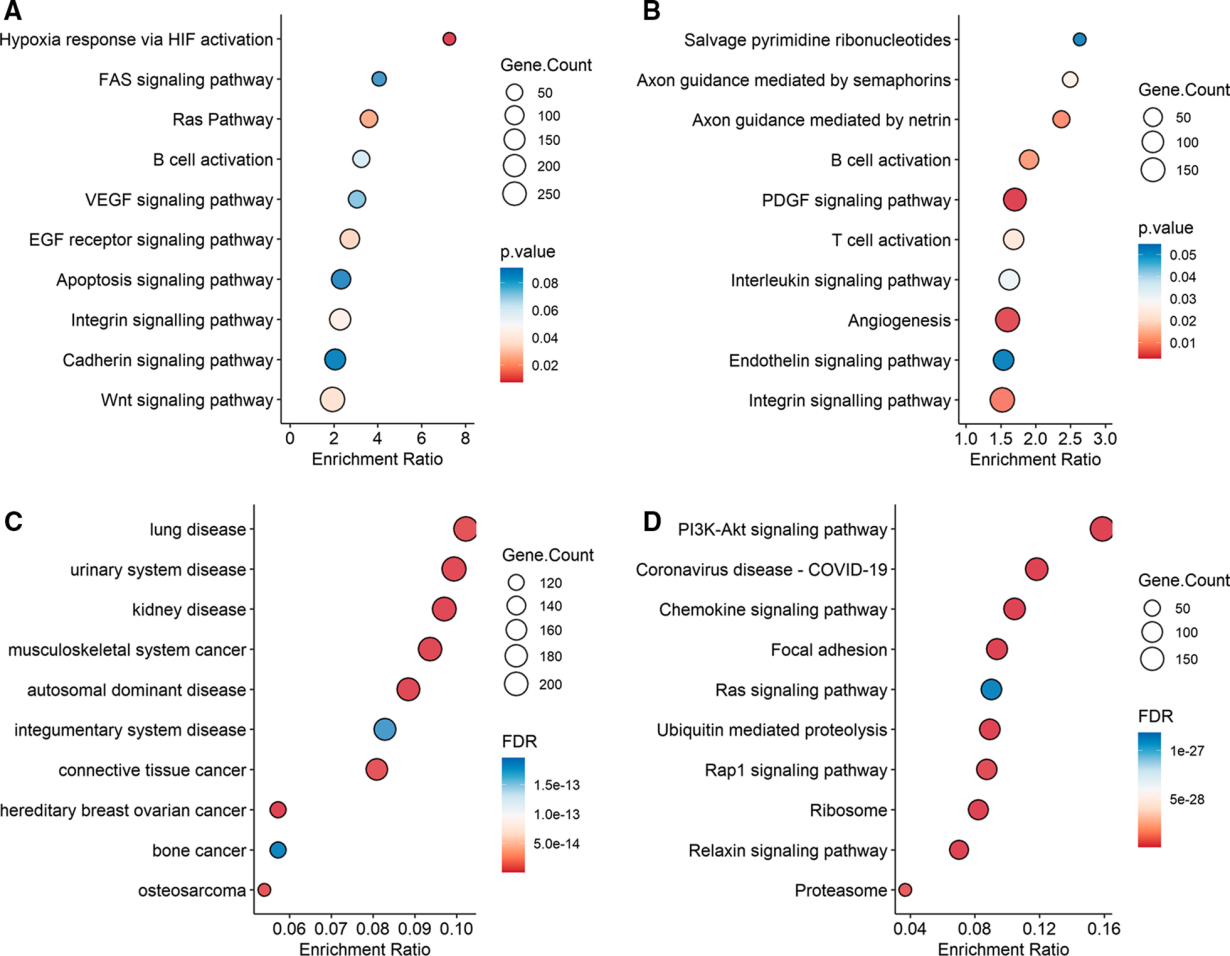
Pathway analyses of TB exposure of the alveolar macrophages and alveolar T cells. The gene count is presented as circles, increasing with elevating number of gene count. The significance level is presented as a color scale. A-B. Alveolar macrophages. Overrepresentation analysis using Panther pathways showing the top candidate pathways of the identified DMGs. The dot plot is demonstrating the most relevant pathways for the alveolar macrophages (**A**) and alveolar T cells (**B**) when comparing the controls with TB-exposed/patients. **C**, **D** Pathways for the alveolar macrophages and alveolar T cells generated with DOSE for enriched diseases (**C**), and KEGG pathways for enriched pathways (**D**). 1051 modules identified with MCODE​ and 1584 modules identified with Clique-Sum were used to identify the top 10 enriched disease and enrich pathways. TB, tuberculosis; DMGs, differentially methylated genes

Using the PANTHER Database, we investigated whether the identified DMGs were enriched in known pathways (Fig. 3A, B). For alveolar macrophages, the analysis revealed enrichment in pathways with relevance for TB infection, including hypoxia-inducible factor (HIF)1 − α activation and the Wnt signaling pathway [22–24] (Fig. 3A). For the alveolar T cells, the overrepresentation analysis using PANTHER Database revealed B and T cell activation, PDGF signaling pathway, angiogenesis and interleukin signaling pathways as top candidate pathways (Fig. 3B). The identified DMGs from the Pat, Exp vs. Con comparison were used as seed genes in the analyses and applied to the protein–protein interaction network from STRINGdb, that resulted in module genes. We then performed a disease enrichment analysis and pathway enrichment analysis using the module genes identified by MCODE. For the enriched analysis of diseases, we found lung disease to be a top candidate (Fig. 3C). Enriched pathways based on module genes for the alveolar macrophages and alveolar T cells included PI3K-Akt, Ras and Chemokine (Fig. 3D).

### Comparisons across cell populations reveals the existence of a common DNA methylome-based biosignature in mycobacteria-exposed immune cells

**Fig. 4:**
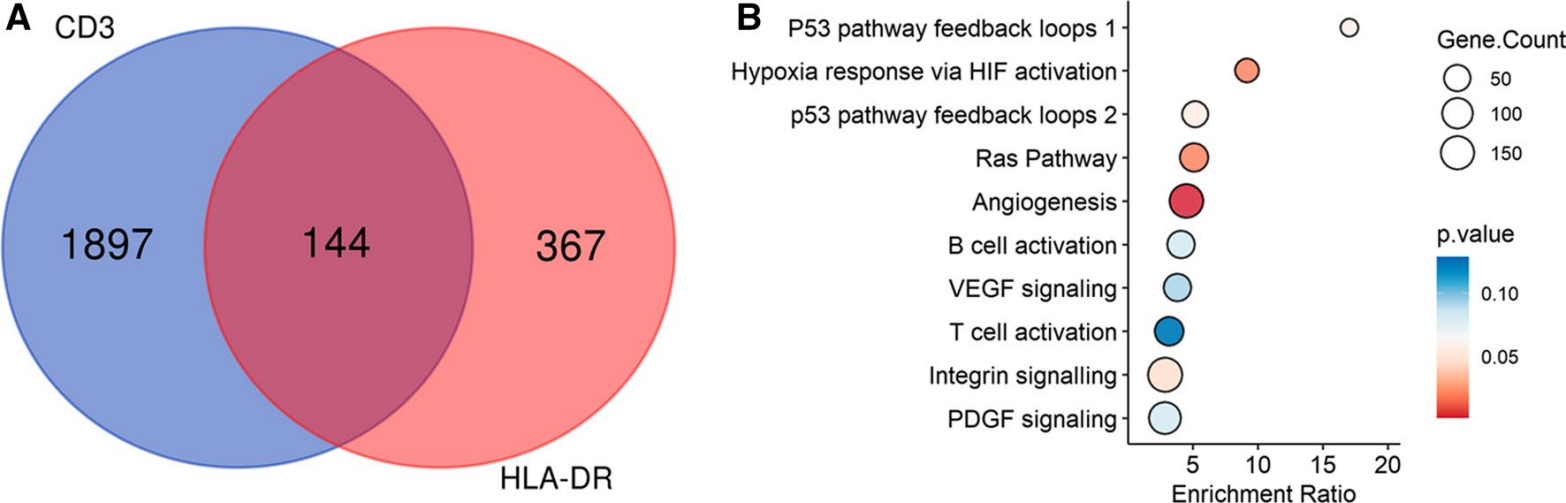
Overlapping DMGs of TB exposure derived from the alveolar macrophages and T cells. **A** Venn plot showing 144 overlapping TB exposure-specific DMGs between the alveolar macrophages and alveolar T cell. FDR < 0.05 and MMD > 0.1. **B** Overrepresentation analysis generated with the PANTHER database of DMGs between the alveolar macrophages and alveolar T cells. DMC, differentially methylated CpG-site; MMD, mean methylation difference; FDR, False Discovery Rate

Given the fact that the interaction between mycobacteria and eukaryotes is evolutionary ancient, we predicted that highly conserved pathways exist that are common among the studied cell populations. Comparing the identified DMGs from the alveolar macrophages and alveolar T cells in a Venn analysis, we discovered 8 common DMGs (MMD > 0.2) (Additional file 5: Fig. S3) and 144 DMGs with reduced stringency (MMD > 0.1) (Fig. 4A). Among the 144 common DMGs between both cell types in this study, separating the Pat, Exp and Con groups we found FBRSL1 (Additional file 9: Table S4). For common DMGs between the alveolar macrophages and alveolar T cells the overrepresentation analysis revealed hypoxia response via HIF-activation, the Ras pathway and VEGF signaling pathway as important pathways (Fig. 4B). We expanded the Venn analyses to include data from our previous work on BCG vaccine-induced DMGs that correlated with enhanced mycobacterial control [4], as natural exposure to TB and BCG vaccination both represent in vivo encounters between mycobacteria and host immune cells. Even though the routes of mycobacterial exposure differ profoundly in these settings, a set of 19 DMGs could be identified as overlapping between our previous BCG study (studying macrophages isolated from peripheral blood) and all cell populations studied here (Additional file 6: Fig. S4), suggesting that a highly conserved epigenetic response to mycobacterial challenge exists.

### Differential DNA methylation analysis identified DMCs to distinguish IGRA positive from IGRA negative subjects

**Fig. 5:**
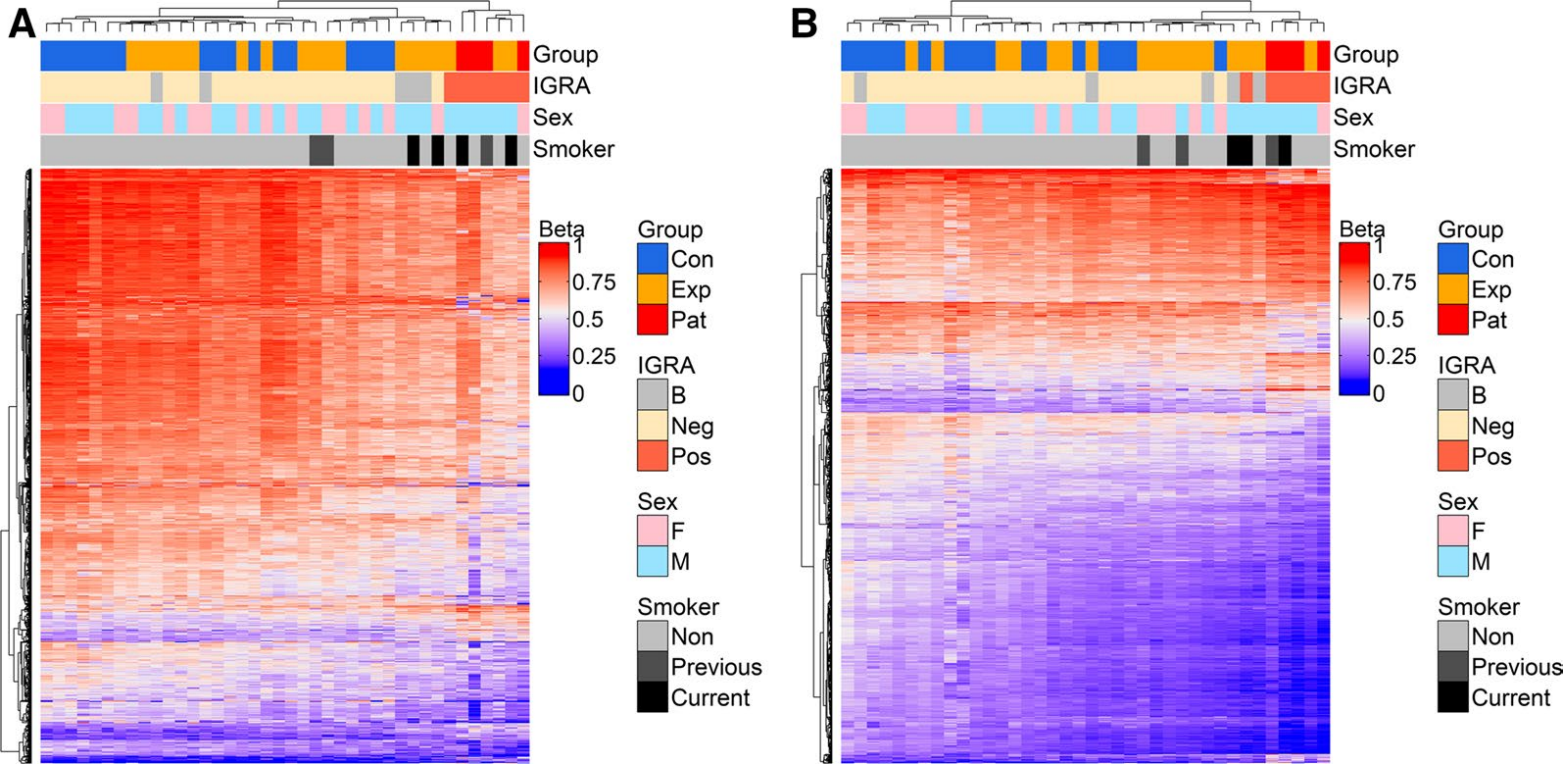
DNAm signatures of IGRA status. Heatmap analysis revealing a distinct DNA methylation pattern of the IGRA positives versus the IGRA negatives in alveolar macrophages (**A**) and alveolar T cells (**B**). The borderline positive samples have been added to the analysis. The heatmap is plotted from the absolute *β*-values of the top DMCs differentiating the groups. Cluster dendrogram is calculated using the Euclidean distance method. DNAm, DNA methylation; IGRA, interferon-gamma release assay; DMC, differentially methylated CpG-site; Con, control group; Exp, TB-exposed; Pat, TB patients; B, borderline positive; F, female; M, male; ND, no data

To determine whether IGRA status can be distinguished based on the methylation pattern, we compared the alveolar macrophage and alveolar T cell DNA methylome data obtained from the IGRA positive and negative subjects. With the *p*-value_BH_ < 0.05 and MMD > 0.1, the analysis revealed 1315 DMCs and 785 DMGs (Fig. 5A; Additional file 9: Table S5) for the alveolar macrophages and 1491 DMCs and 855 DMGs for the alveolar T cells (Fig. 5B; Additional file 9: Table S6). After demonstrating that the IGRA positive and negative groups had distinct DNA methylation traits, we performed a heatmap analysis based on the top DMCs of the DNA methylation analysis that showed that the IGRA positive group clustered into one homogenous group for both the alveolar macrophages and alveolar T cells (Fig. 5A, B). The pathway analyses of the IGRA related genes for the alveolar macrophages and alveolar T cells are shown in Additional files 7 and 8: Figure S5A and S5B.

## Discussion

The epigenetic response to infection is poorly studied in humans, and the few studies that exist focus on individuals who have ongoing disease symptoms and conclude that the observed alterations in epigenetic patterns are a pathological event [25]. However, in recent years, several studies performed in fish points toward a contribution of altered DNA methylation patterns to resistance against infections [26, 27]. We previously demonstrated that a distinct DNA methylation pattern in immune cells of BCG-vaccinated subjects correlated with increased capacity of monocyte-derived macrophages to kill *M. tuberculosis* [4]. We have also described that subjects, who IGRA converted during the course of the study, displayed a differential DNA methylation pattern before IGRA conversion [28]. In this study, we present data suggesting that exposure to TB generates a distinct DNAm signature in pulmonary immune cells. The signature was found not only in those with TB infection (TB patients or positive IGRA), but also in individuals, who are exposed to TB but IGRA negative. The finding that healthy, TB-exposed individuals also carry the signature opens the possibility that the epigenetic alterations reflect a host-beneficial reprogramming of the immune mechanisms rather than being induced by *M. tuberculosis* as a step to evade the immune defense. The way TB disease is viewed has shifted from a binary perspective (latent or active TB) to be considered a spectrum of disease ranging from early clearance, latent infection to various presentations of active TB [29–32]. 32% of the TB-exposed subjects in this study and 11% of the controls had a positive IGRA result, corresponding to the declared exposure to TB. One theory why one healthy control with a borderline-positive result, indicating host immune responses against *M. tuberculosis*, aligned with the rest of the healthy controls, is that the subject was exposed to TB a long time ago, while the IGRA positive TB-exposed subjects had a more recent exposure. A diagnostic tool to specify who will clear the bacteria or develop active TB upon TB exposure and to define the risk of LTBI conversion to active disease would aid in contact tracing and treatment decision making [33]. Attempts have previously been made to identify patients with LTBI with a high risk of developing active TB using RNA-sequencing, but with lacking confirmatory longitudinal data [34]. The DNAm signatures of TB and TB exposure found in this study could be used to determine who has recently been exposed to TB in a contact tracing context. Further studies need to confirm the DNAm patterns in a larger cohort and investigate longitudinally whether the signature seen in the TB-exposed subjects reflects a beneficial response or risk of becoming ill with TB. The TB patients formed a separate group based on their DNAm pattern, but some differences could be found between the Lima and Linköping cohort (drug-resistant TB and drug-sensitive TB, respectively), suggesting that the type of TB-treatment and different treatment responses are also reflected in the DNA methylome. In line with the fact that macrophages constitute the main niche for mycobacterial replication, the strongest enrichment of DNAm changes was observed in the alveolar macrophage population. The pathways identified to be enriched in the alveolar macrophage population for both TB exposure and IGRA status have been described in the context of trained immunity, BCG exposure and TB. For example, activation of Hypoxia-Inducible Factor 1 α and glycolysis pathways are hallmarks of macrophages that have undergone the epigenetic changes reflective of trained immunity [35], which is induced in myeloid cells upon BCG-stimulation [36, 37]. VEGF-release by macrophages has been shown to recruit immune cells during granuloma formation [38]. Several studies have ascribed Wnt pathways immunomodulating functions and induction during *M. tuberculosis* infection (reviewed in [39]). The Ras pathway appeared in the pathway analysis of the alveolar macrophages, alveolar T cells and the pathway analysis of the common DMCs between the cell types. In a recent study where we found that a distinct DNA methylome signature characterized IGRA negative subjects, who later presented with a positive IGRA result [28], we showed that the Ras pathway is of importance in IGRA conversion [28]. Also, the PI3K-Akt pathways appeared for both the alveolar T cells and the common pathway analysis. The top pathway for the alveolar macrophages using MCODE was lung disease, indicating that genes of importance for lung inflammation are affected upon TB exposure. In the Venn diagram, when comparing the DMGs from this study with DMGs from our previous publication [4] we found 17 common genes for the alveolar macrophages, and 266 common genes for the alveolar T cells. FBRSL1 was found as a common DMG between alveolar macrophages and alveolar T cells. FBRSL1 has been shown to be an important gene in a recent published study on TB related DNA methylation biomarkers in peripheral blood [40]. BCG vaccination has convincingly been shown to induce heterologous immunity protecting against childhood mortality from other causes than TB [41, 42]. Based on our finding that natural TB exposure and BCG vaccination trigger similar epigenetic changes, and that the involved pathways overlap with those found in trained immunity, we propose the hypothesis that a “beneficial exposure” to TB exists, which protects against other infections through heterologous immunity. Along the same line, it has been shown that a substantial fraction of individuals exposed to TB can be defined as ‘early clearers’, since they remain tuberculin skin test or IGRA negative, suggesting effective eradication of the infection [43]. A limitation of the present study is that no data on bacteria clearance or antimycobacterial efficacy of the cells is available, therefore no conclusion can be drawn on the correlation between the epigenetic changes and their effect.

## Conclusions

Our study suggests that DNA methylome analysis is a promising approach to study the spectrum of TB. Our results affirm previous work by us and others on mycobacteria-induced epigenetic changes in immune cells. Longitudinal studies of patients with TB during treatment are needed that cover DNA methylation changes of alveolar macrophages and alveolar T cells before the time point of IGRA conversion over the development of active TB and during TB treatment until cure. The results will allow dissection of the unique DNAm signatures for each state of TB and could be further developed as precision tools for clinical TB diagnosis, contact-tracing, decision-making in LTBI infected subjects and TB treatment monitoring.

## Methods

### Study design and participants

Patients with pulmonary TB, healthy participants with a history of TB-exposure and healthy controls, with an age ranging from 18 to 53 years, were enrolled at Linköping University Hospital and Linköping University, respectively. To determine epigenetic changes in the immune cells in TB-exposed individuals, we recruited subjects enrolled in a TB contact tracing at Linköping University Hospital, Sweden, according to standard contact tracing routines (household contacts and persons with > 8 h of interaction with the index case). We included two index cases and five contact tracing subjects who were ethnicity-matched (Table 1). The patients were diagnosed with drug-sensitive pulmonary TB. In a secondary inclusion of subjects we directed our focus towards Peru, which has a higher prevalence of TB than Sweden. Peruvian health care workers and medical students have an increased risk to acquire a TB infection [44, 45]. Because we were interested in studying DNAm changes upon TB exposure, subjects were recruited from the Hospital Nacional Cayetano Heredia in Lima, Peru, and controls were recruited from Universidad Peruana Cayetano Heredia, also in Lima. Following oral and written consent, two patients with drug-resistant TB, 14 occupational-exposed subjects and 12 controls (18–40 years of age) were included in the study. We obtained induced sputum from each participant and used an established protocol for the separate isolation of alveolar macrophages and alveolar T cells.

### Sputum induction and pulmonary immune cell isolation

Induced sputum is a well-tolerated, non-invasive method to collect cells from the respiratory compartment after inhalation of a hypertonic saline solution. The procedure of sputum induction takes approximately 30 min and is both cost effective and safe with minimal clinical risks [46]. Sputum specimens were collected as described by Alexis et al*.* [47], with the following modification: premedication with an adrenergic β2-agonist, salbutamol (Ventoline, 1 ml, 1 mg/ml) was administrated before the inhalation of hypertonic saline, using a nebulizer (eFlow, PARI), in Sweden but not in Peru, following local protocols. The subsequent steps of sputum processing were adopted from Alexis et al*.* [48] and Sikkeland et al*.* [20]. The alveolar macrophages and alveolar T cells were isolated using superparamagnetic beads coupled with anti-human CD3 and Pan Mouse IgG antibodies and HLA-DR/human MHC class II antibodies (Invitrogen Dynabeads, ThermoFisher, cat no. 11041 and 14-9956-82, respectively). An initial positive selection was done with CD3 beads followed by a positive HLA-DR selection. Bead-coating and cell isolation were performed according to manufacturer’s protocol.

### DNAm sequencing and statistical analyses

DNA from the alveolar macrophages and alveolar T cells were extracted using the AllPrep DNA/RNA Mini Kit (Qiagen, Hilden, Germany) according to the manufacturer’s instructions. Genome-wide DNA methylation analysis of the DNA from alveolar macrophages and alveolar T cells was performed using the Infinium Human Methylation 450 K Bead Chip array (Illumina) as per the manufacturer’s instructions. The technique is based on quantitative genotyping of C/T polymorphism which is generated by DNA bisulfite conversion and allows for the assessment of over 450 000 methylation sites within the whole genome. DNA was converted and amplified and subsequently fragmented and hybridized to the Infinium HumanMethylation450 Bead Chip. The procedure was conducted at the Bioinformatics and Expression analysis Core facility at the Karolinska Institute, Sweden. The methylation profiles from the HumanMethylation450K BeadChip analysis for each cell type were analyzed from the raw IDAT files in R (v4.0.2) using the *minfi* (v1.36.0) with subset-quantile within array (SWAN) normalization [49, 50]. *ChAMP* [51] (v2.19.3). The data were pre-processed in several steps, following probes were filtered out: (1) probes below the detection *p*-value (> 0.01), (2) non-CpG probes, (3) multi-hit probes, and (4) all probes of X and Y chromosomes. Batch effects were corrected using *ComBat* from the *SVA* package [21] (v3.38.0) in *ChAMP* (v2.19.3). Identified sources of variation that were still present upon SVD correction were included as co-variates in the models. Differential methylation analysis was performed with the linear modeling (lmFit) using the *limma* package [52] (v3.46.0) in a contrast matrix of the TB-exposed and TB-non-exposed (Control) individuals. All differentially methylated CpGs (DMCs) were considered significant at the corrected *p*-value_BH_ < 0.05 and mean methylation difference (MMD) > 0.2. The DMCs were mapped to their corresponding genes as differentially methylated genes (DMGs) using the hg19 annotations.

### Unsupervised cluster analysis

Hierarchical clustering of the all TB-exposed and control individuals was performed with the normalized, batch corrected *β *values obtained after the data filtration in each cell type individually. The distance was calculated using the Euclidean distance matrix. The *dendextend* [52] (v1.14.0) and *ape* [53] (v5.4-1) packages in R were used to construct the horizontal hierarchical plots from the three different cell populations using the *hclust* and *dendrogram* functions.

### Structural annotations

The *EnhancedVolcano* package [54] (v1.8.0) was used to generate the individual volcano plots from all cell populations. The heatmaps were generated from the filtered DMGs with their respective CpGs for each cell type using the *ComplexHeatmap* (v2.6.2) package [55]. The clustering dendrogram in heatmaps were plotted using the Euclidean distance matrix. The DMCs identified in all four comparisons (Exp vs Con, Pat vs Con, Pat vs Exp, Exp + Pat vs Con), were merged/combined to generate a single heatmap. A mean methylation difference (MMD) of > 0.2 was used for the groupwise analyses. MMD > 0.1 was used for the comparison of IGRA positives vs negatives. For the IGRA status analysis, the TB patients were regarded as IGRA positives and included in the IGRA positive group.

### Pathway overrepresentation and enrichment analyses

The identified DMGs (relaxed cutoffs of MMD > 0.1) in all the above comparisons were used for PANTHER pathway over-representation analyses using the WEB-based Gene SeT AnaLysis Toolkit (WebGestalt) webserver (v2022). The FDR in the pathway analysis is BH adjusted *p*-values. We used nominal *p*-values (significance level set to *p* < 0.05) in case FDR correction was too stringent. Top ten significantly enriched pathways were displayed in dot plots generated in R using ggplot2 package (version 3.3.3).

Furthermore, the graph clustering algorithm MCODE and Clique Sum (Clique Susceptibility Module algorithm) in Modifier package was used to identify molecular complexes and create a large disease modules. The identified DMGs were used as seed genes and applied to the protein–protein interaction network from STRINGdb (confidence score > 0·7), that resulted in module genes. For the module genes, we used package DOSE (Disease Ontology Semantic and Enrichment analysis) to identify the enriched diseases (Fig. 3C) and using *clusterProfiler* [56] (v3.18.1), we performed KEGG pathway [57] enrichment analysis (Fig. 3D). To enhance the visualization and better understanding of the enrichment result, ggplot2 package was used to generate the dot plots.

### Venn and overlap analyses

Venn analyses were performed to detect the DMGs overlapping between cell populations and a previous publication. We constructed the Venn diagrams by using ugent Venn diagram generator (http://bioinformatics.psb.ugent.be/webtools/Venn/) and in house R script to compare the DMGs.

### Statistical analyses

All differences with a *p*-value_BH_ < 0.05 were considered significant if not otherwise stated. We calculated family-wise error rate (FWER) using the BH correction method. All analyses were performed in R (v4.0.2) with the mentioned packages.

## Supplementary Information

**Additional file 1.** Figure legends Supplementary figures.

**Additional file 2. Supplemental figure S1**. Singular Value Decomposition (SVD) analysis of subject characteristics. We found no significant difference between the Pat, Exp and Con groups regarding BMI, smoking, age and sex. For asthma, IGRA and batch (also reflecting country; Peru or Sweden) we found significant differences in the DNA methylomes. Pat, TB patients; Exp, TB-exposed; Con, control group; BMI, body mass index; IGRA, interferon-gamma release assay.

**Additional file 3. Supplemental figure S2**. MDS plot showing the origin of the sample (Lima or Linköping). Alveolar macrophage (A) and alveolar T cell (B) samples from the Lima cohort are presented as dots and samples from the Linköping cohort are presented as triangles. The patients are shown in red. The Lima patients (n=2) have drug-resistant TB and the Linköping patients (n=2) have drug-sensitive TB. MDS, multidimensional scaling.

**Additional file 4. Supplemental figure S2**. MDS plot showing the origin of the sample (Lima or Linköping). Alveolar macrophage (A) and alveolar T cell (B) samples from the Lima cohort are presented as dots and samples from the Linköping cohort are presented as triangles. The patients are shown in red. The Lima patients (n=2) have drug-resistant TB and the Linköping patients (n=2) have drug-sensitive TB. MDS, multidimensional scaling.

**Additional file 5. Supplemental figure S3**. Overlapping DMGs derived from the alveolar macrophages and T cells. FDR< 0.05 and MMD > 0.2. DMGs, differentially methylated genes; FDR, False Discovery Rate; MMD, mean methylation difference.

**Additional file 6. Supplemental figure S4**. Overlapping genes with our previous study*. We found 19 common genes for both alveolar macrophages and lymphocytes. *D. Verma et al, 2017.

**Additional file 7. Supplemental figure S5**. Pathway analysis of IGRA status based on DMGs found between the IGRA positive and IGRA negative participants. A-B. Pathways of IGRA status in alveolar macrophages (A), based on 785 DMGs, and in alveolar T cells (B), based on 855 DMGs. IGRA, interferon-gamma release assay; DMGs, differentially methylated genes.

**Additional file 8. Supplemental figure S5**. Pathway analysis of IGRA status based on DMGs found between the IGRA positive and IGRA negative participants. A-B. Pathways of IGRA status in alveolar macrophages (A), based on 785 DMGs, and in alveolar T cells (B), based on 855 DMGs. IGRA, interferon-gamma release assay; DMGs, differentially methylated genes.

**Additional file 9. Supplemental table S1**. Differentially methylated CpG sites (DMCs) from analyses in alveolar macrophages for TB status. Merged list of DMCs from Pat, Exp and Con contrasts using absolute MMD > 0.2 and Ajd.p-val < 0.05.** Supplemental table S2**. Differentially methylated CpG sites (DMCs) from analyses in alveolar T cells for TB status. Merged list of DMCs from Pat, Exp and Con contrasts using absolute MMD > 0.2 and Ajd.p-val < 0.05.** Supplemental table S3**. Differentially methylated CpG sites (DMCs) identified between Linköping and Lima patient samples (n= 2+2) in Alveolar T cells with Adj.p-val <0.1. (No DMCs identified in HLA-DR).** Supplemental table S4**. Common Differentially methylated Genes (DMGs) from analyses in alveolar macrophages and T cells for TB status. Using relaxed cutoffs MMD > 0.1 and Ajd.p-val < 0.05.** Supplemental table S5**. Differentially methylated CpG sites (DMCs) identified in alveolar macrophages for IGRA status with MMD >0.1, Ajd.p-val < 0.05.** Supplemental table S6**. Differentially methylated CpG sites (DMC) identified in alveolar T cells for IGRA status with MMD >0.1, Ajd.p-val< 0.05.

## Data Availability

The datasets generated and/or analysed during the current study are not publicly available due to intellectual property restrictions but are available from the corresponding author on reasonable request.
