# Supplementary material for: The spectrum of tuberculosis described as differential DNA methylation patterns in alveolar macrophages and alveolar T cells
Source: Clin Epigenetics. 2022 Dec 17;14:175. doi: 10.1186/s13148-022-01390-9 (PMC9758029; doi:10.1186/s13148-022-01390-9)
Supplement: Supplementary file 1 — Additional file 1. Figure legends Supplementary figures. [file 13148_2022_1390_MOESM1_ESM.docx]

**Figure legends Supplementary figures**

Additional file 2: **Supplemental figure S1.** Singular Value Decomposition (SVD) analysis of subject characteristics. We found no significant difference between the Pat, Exp and Con groups regarding BMI, smoking, age and sex. For asthma, IGRA and batch (also reflecting country; Peru or Sweden) we found significant differences in the DNA methylomes. Pat, TB patients; Exp, TB-exposed; Con, control group; BMI, body mass index; IGRA, interferon-gamma release assay.

Additional file 3 and 4: **Supplemental figure S2.** MDS plot showing the origin of the sample (Lima or Linköping). Alveolar macrophage (A) and alveolar T cell (B) samples from the Lima cohort are presented as dots and samples from the Linköping cohort are presented as triangles. The patients are shown in red. The Lima patients (*n*=2) have drug-resistant TB and the Linköping patients (*n*=2) have drug-sensitive TB. MDS, multidimensional scaling.

Additional file 5: **Supplemental figure S3.** Overlapping DMGs derived from the alveolar macrophages and T cells. FDR< 0.05 and MMD > 0.2. DMGs, differentially methylated genes; FDR, False Discovery Rate; MMD, mean methylation difference.

Additional file 6: **Supplemental figure S4.** Overlapping genes with our previous study*. We found 19 common genes for both alveolar macrophages and lymphocytes. *D. Verma et al, 2017.

Additional file 7 and 8: **Supplemental figure S5.** Pathway analysis of IGRA status based on DMGs found between the IGRA positive and IGRA negative participants. A-B. Pathways of IGRA status in alveolar macrophages (A), based on 785 DMGs, and in alveolar T cells (B), based on 855 DMGs. IGRA, interferon-gamma release assay; DMGs, differentially methylated genes.

**Table legends Supplementary tables**

Additional file 9: **Supplemental table S1.** Differentially methylated CpG sites (DMCs) from analyses in alveolar macrophages for TB status. Merged list of DMCs from Pat, Exp and Con contrasts using absolute MMD > 0.2 and Ajd.p-val < 0.05.

Additional file 9: **Supplemental table S2.** Differentially methylated CpG sites (DMCs) from analyses in alveolar T cells for TB status. Merged list of DMCs from Pat, Exp and Con contrasts using absolute MMD > 0.2 and Ajd.p-val < 0.05.

Additional file 9: **Supplemental table S3.** Differentially methylated CpG sites (DMCs) identified between Linköping and Lima patient samples (*n*= 2+2) in Alveolar T cells with Adj.p-val <0.1. (No DMCs identified in HLA-DR)

Additional file 9: **Supplemental table S4.** Common Differentially methylated Genes (DMGs) from analyses in alveolar macrophages and T cells for TB status. Using relaxed cutoffs MMD > 0.1 and Ajd.p-val < 0.05.

Additional file 9: **Supplemental table S5.** Differentially methylated CpG sites (DMCs) identified in alveolar macrophages for IGRA status with MMD >0.1, Ajd.p-val < 0.05.

Additional file 9: **Supplemental table S6**. Differentially methylated CpG sites (DMC) identified in alveolar T cells for IGRA status with MMD >0.1, Ajd.p-val< 0.05.
