# Supplementary figures and images for: The spectrum of tuberculosis described as differential DNA methylation patterns in alveolar macrophages and alveolar T cells
Source: Clin Epigenetics. 2022 Dec 17;14:175. doi: 10.1186/s13148-022-01390-9 (PMC9758029; doi:10.1186/s13148-022-01390-9)

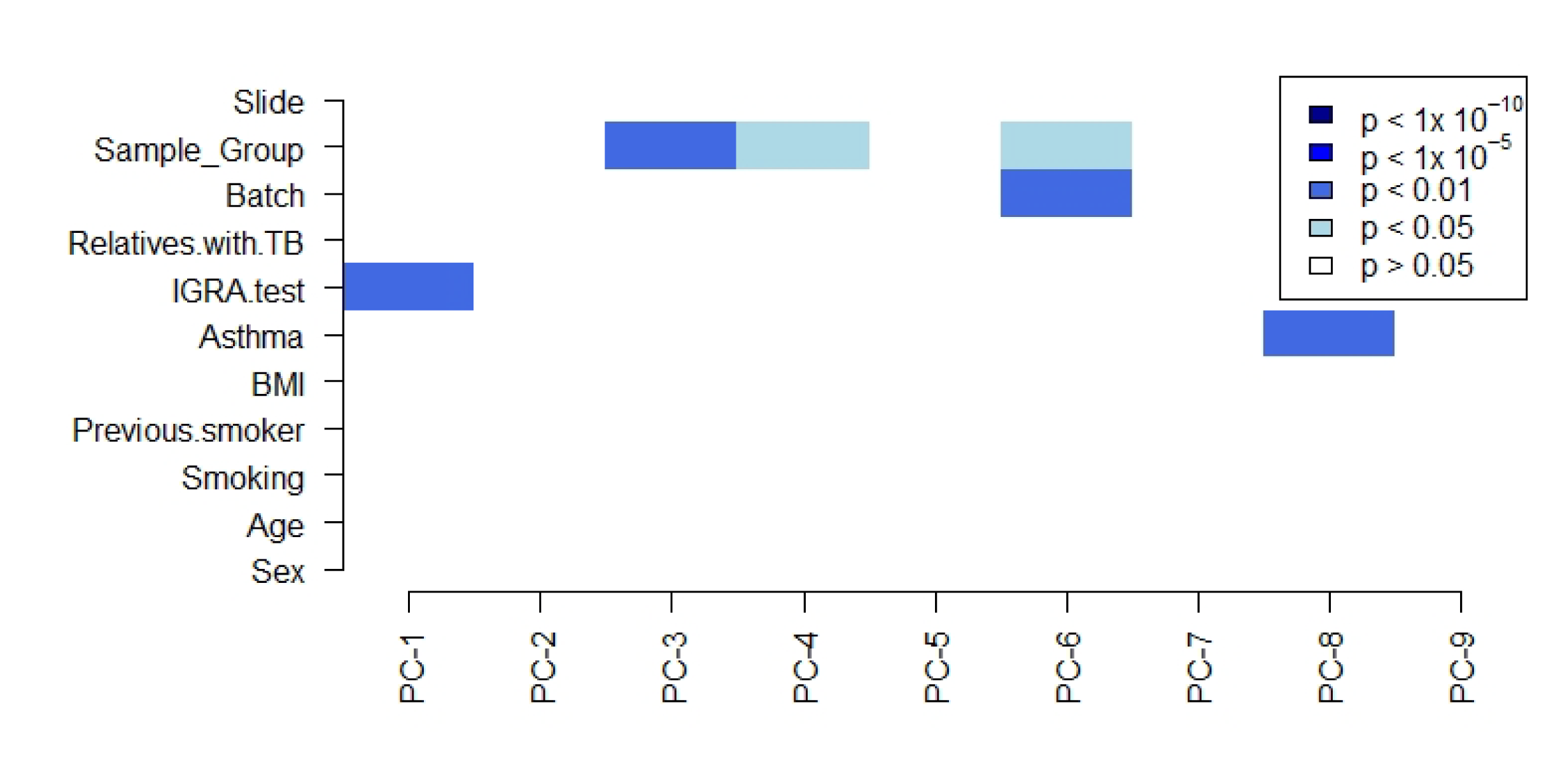

Supplement: Supplementary file 2 — Additional file 2. Supplemental figure S1. Singular Value Decomposition (SVD) analysis of subject characteristics. We found no significant difference between the Pat, Exp and Con groups regarding BMI, smoking, age and sex. For asthma, IGRA and batch (also reflecting country; Peru or Sweden) we found significant differences in the DNA methylomes. Pat, TB patients; Exp, TB-exposed; Con, control group; BMI, body mass index; IGRA, interferon-gamma release assay. [file 13148_2022_1390_MOESM2_ESM.tif]

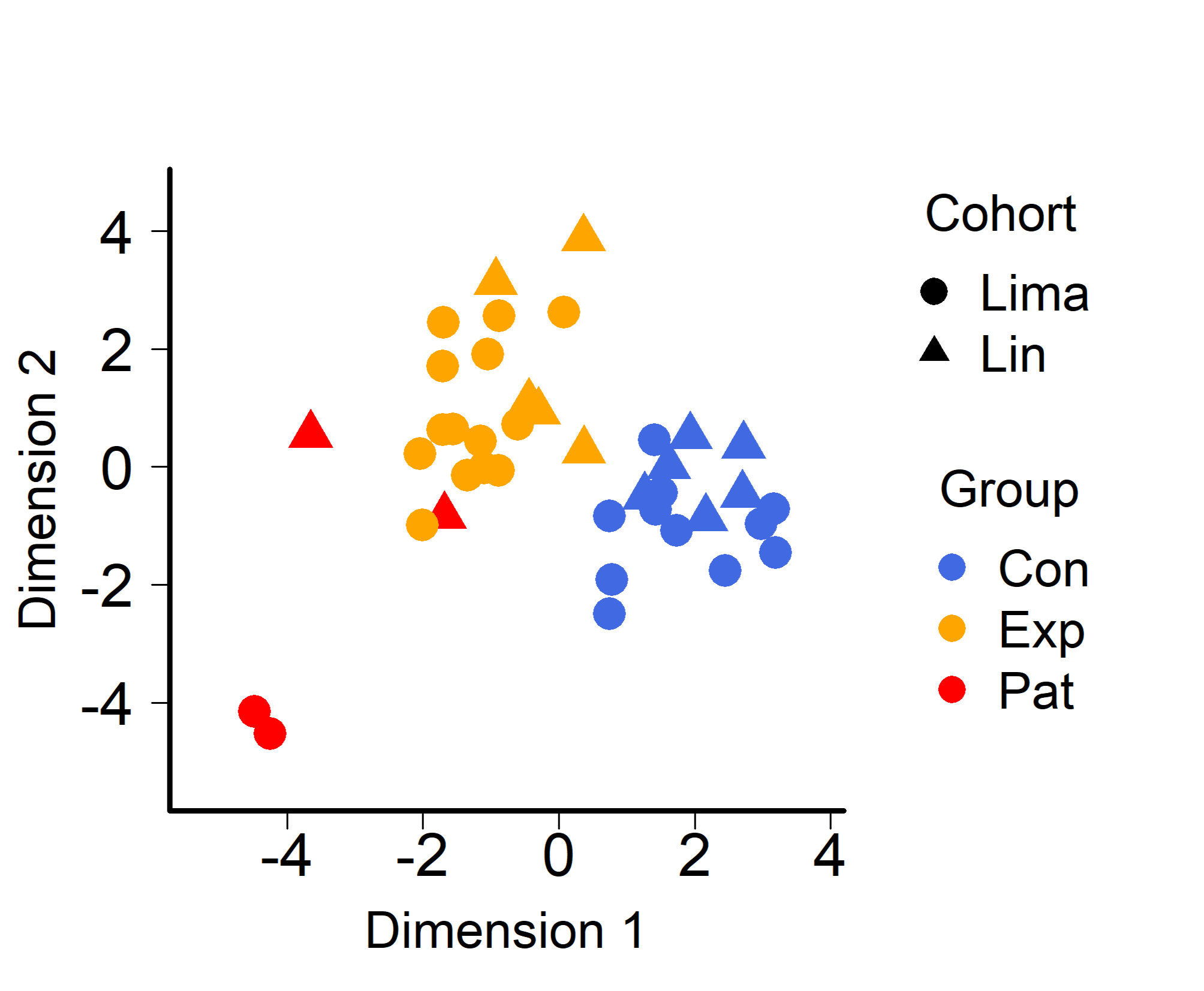

Supplement: Supplementary file 3 — Additional file 3. Supplemental figure S2. MDS plot showing the origin of the sample (Lima or Linköping). Alveolar macrophage (A) and alveolar T cell (B) samples from the Lima cohort are presented as dots and samples from the Linköping cohort are presented as triangles. The patients are shown in red. The Lima patients (n=2) have drug-resistant TB and the Linköping patients (n=2) have drug-sensitive TB. MDS, multidimensional scaling. [file 13148_2022_1390_MOESM3_ESM.tif]

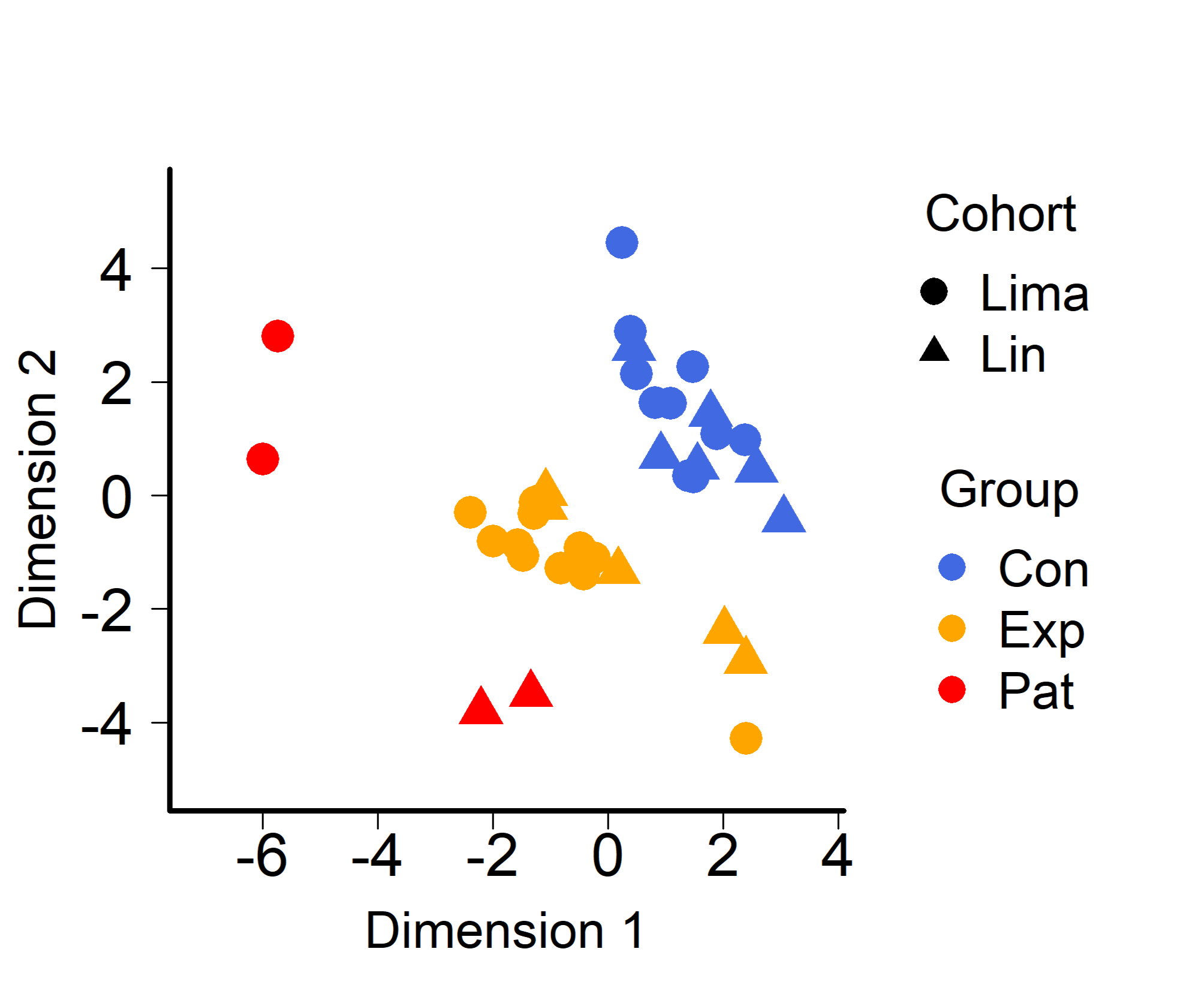

Supplement: Supplementary file 4 — Additional file 4. Supplemental figure S2. MDS plot showing the origin of the sample (Lima or Linköping). Alveolar macrophage (A) and alveolar T cell (B) samples from the Lima cohort are presented as dots and samples from the Linköping cohort are presented as triangles. The patients are shown in red. The Lima patients (n=2) have drug-resistant TB and the Linköping patients (n=2) have drug-sensitive TB. MDS, multidimensional scaling. [file 13148_2022_1390_MOESM4_ESM.tif]

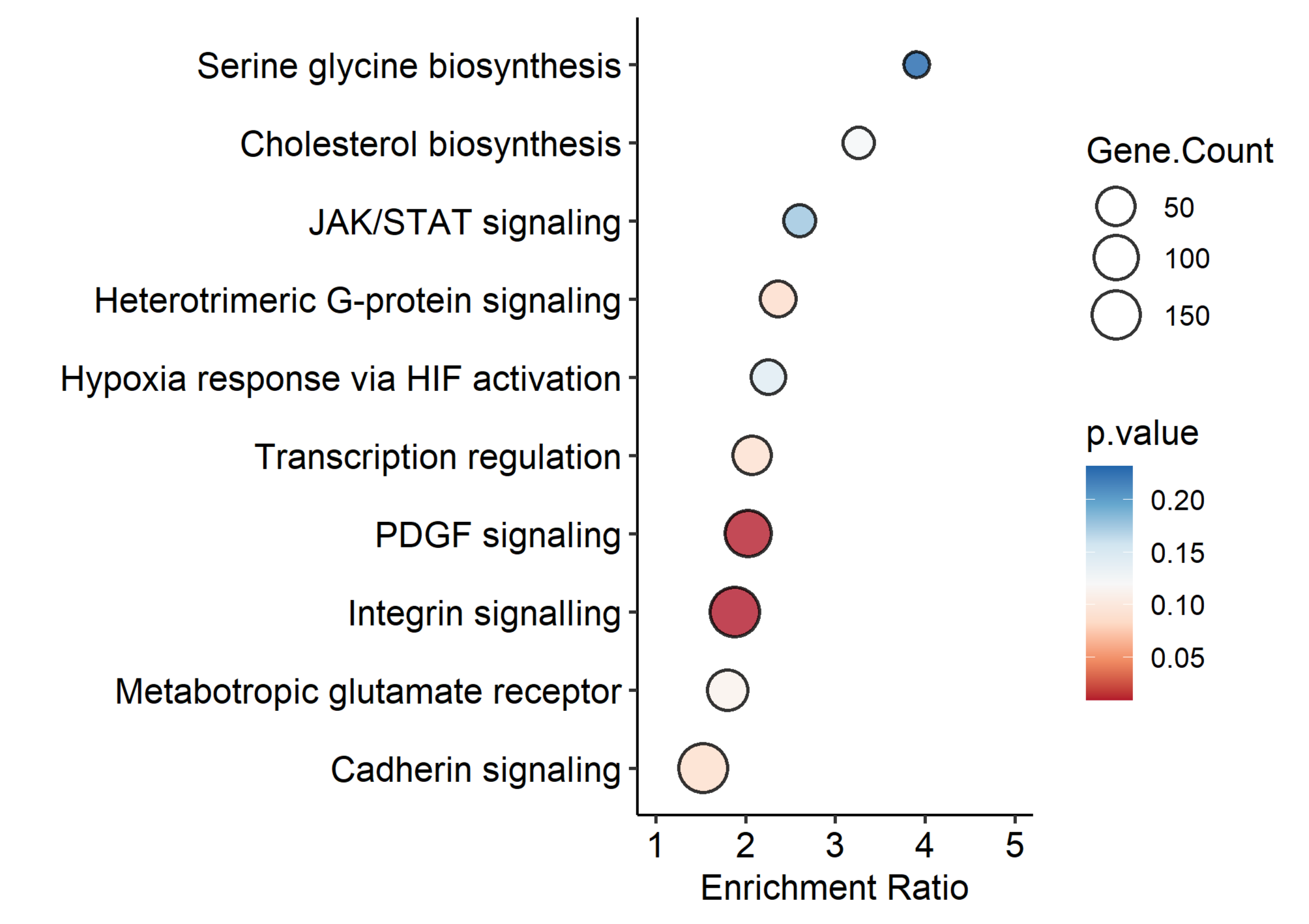

Supplement: Supplementary file 7 — Additional file 7. Supplemental figure S5. Pathway analysis of IGRA status based on DMGs found between the IGRA positive and IGRA negative participants. A-B. Pathways of IGRA status in alveolar macrophages (A), based on 785 DMGs, and in alveolar T cells (B), based on 855 DMGs. IGRA, interferon-gamma release assay; DMGs, differentially methylated genes. [file 13148_2022_1390_MOESM7_ESM.tif]

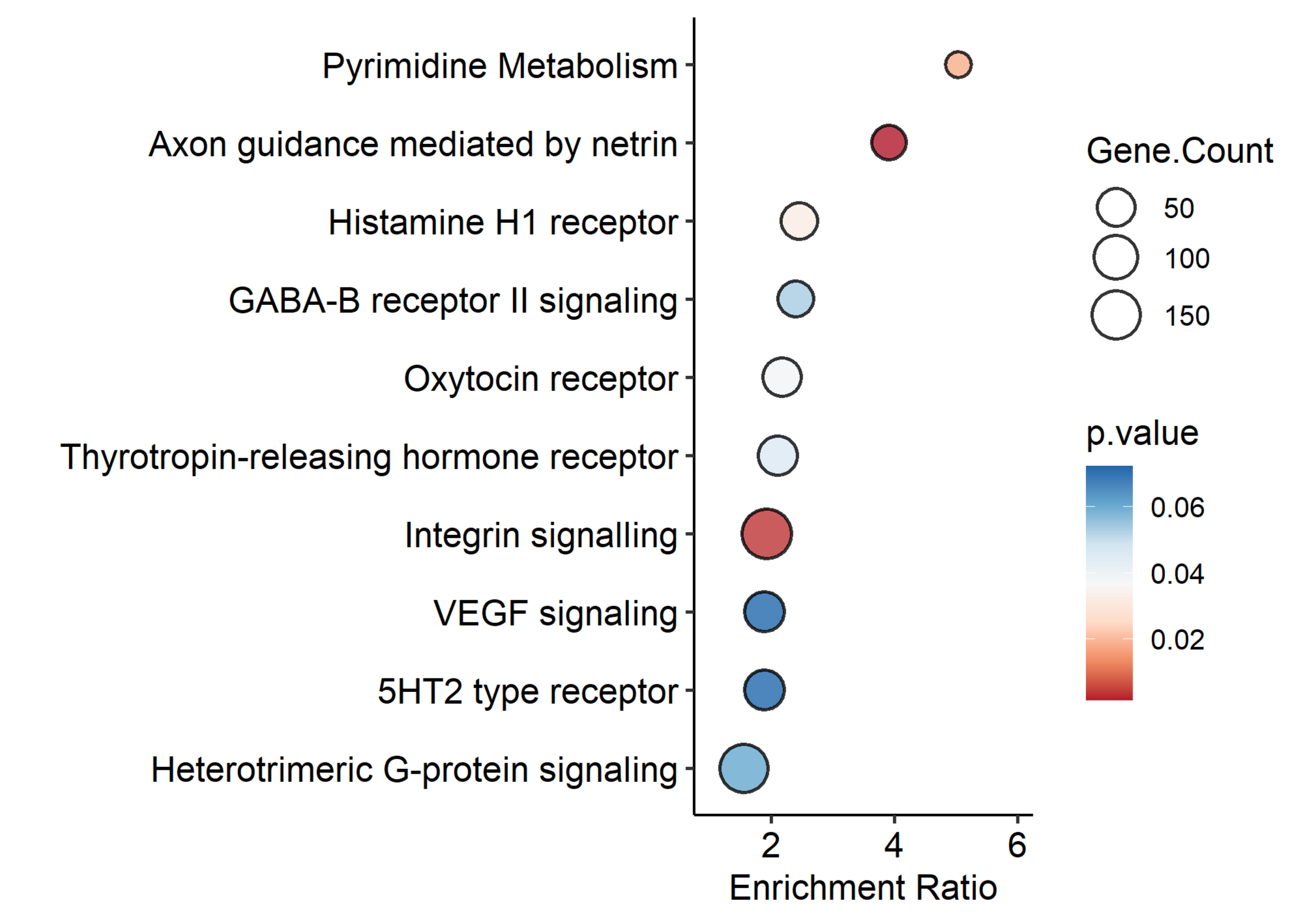

Supplement: Supplementary file 8 — Additional file 8. Supplemental figure S5. Pathway analysis of IGRA status based on DMGs found between the IGRA positive and IGRA negative participants. A-B. Pathways of IGRA status in alveolar macrophages (A), based on 785 DMGs, and in alveolar T cells (B), based on 855 DMGs. IGRA, interferon-gamma release assay; DMGs, differentially methylated genes. [file 13148_2022_1390_MOESM8_ESM.tif]
